# Statin use and the risk of colorectal cancer in a population-based electronic health records study

**DOI:** 10.1038/s41598-019-49877-5

**Published:** 2019-09-19

**Authors:** Gemma Ibáñez-Sanz, Elisabet Guinó, Caridad Pontes, Mª Ángeles Quijada-Manuitt, Luisa C de la Peña-Negro, María Aragón, Marga Domínguez, Lorena Rodríguez-Alonso, Alex Blasco, Ana García-Rodríguez, Rosa Morros, Victor Moreno

**Affiliations:** 1 Programa d’Analítica de Dades en Oncologia, Institut Català d’Oncologia, L’Hospitalet de Llobregat, Spain; 2 Servei d’Aparell Digestiu, Hospital Universitari de Bellvitge, L’Hospitalet de Llobregat, Spain; 3 Grup de Càncer Colorectal, Programa ONCOBELL, Institut d’Investigació Biomèdica de Bellvitge (IDIBELL), Hospitalet de Llobregat, Spain; 4 CIBER Epidemiologia i Salud Pública (CIBERESP), Madrid, Spain; 5 Departament de Farmacologia, de Terapèutica i de Toxicologia, Universitat Autònoma de Barcelona, Barcelona, Spain; 6 Departament de Farmacologia Clínica, Hospital de la Santa Creu i Sant Pau, Barcelona, Spain; 7 Departament de Patologia i Terapèutica Experimental. Unitat Docent Campus de Bellvitge, Universitat de Barcelona, L’Hospitalet de Llobregat, Spain; 8 Servei d’Aparell Digestiu, Hospital de Viladecans, Viladecans, Spain; 9 Fundació Institut Universitari per a la recerca a l’Atenció Primària de Salut Jordi Gol i Gurina (IDIAPJGol), Barcelona, Spain; 10 Servei d’Aparell Digestiu, Hospital de Moisés Broggi, Sant Joan Despí, Spain; 11 Institut Català de la Salut (ICS), Barcelona, Spain; 12 Departament de Ciències Clíniques, Facultat de Medicina i Ciències de la Salut, Universitat de Barcelona, Barcelona, Spain

**Keywords:** Disease prevention, Epidemiology, Colorectal cancer, Risk factors

## Abstract

There is extensive debate regarding the protective effect of 3-hydroxy-3-methylglutaryl-coenzyme A reductase inhibitors (statins) on colorectal cancer (CRC). We aimed to assess the association between CRC risk and exposure to statins using a large cohort with prescription data. We carried out a case-control study in Catalonia using the System for Development of Primary Care Research (SIDIAP) database that recorded patient diseases history and linked data on reimbursed medication. The study included 25 811 cases with an incident diagnosis of CRC between 2010 and 2015 and 129 117 frequency-matched controls. Subjects were classified as exposed to statins if they had ever been dispensed statins. Analysis considering mean daily defined dose, cumulative duration and type of statin were performed. Overall, 66 372 subjects (43%) were exposed to statins. There was no significant decrease of CRC risk associated to any statin exposure (OR = 0.98; 95% CI: 0.95–1.01). Only in the stratified analysis by location a reduction of risk for rectal cancer was observed associated to statin exposure (OR = 0.87; 95% CI: 0.81–0.92). This study does not support an overall protective effect of statins in CRC, but a protective association with rectal cancer merits further research.

## Introduction

Colorectal cancer (CRC) is the third most common cancer worldwide^1^ and its incidence is still rising in many low and middle income countries^2^. Focus on primary prevention and screening is necessary in order to reduce the incidence and mortality of this cancer. Although lifestyle risk factors have been identified in CRC^3^, randomized trials have failed to show a reduction of adenomas recurrence with diet^4–6^ or dietary supplements^7,8^. A large body of evidence has shown that nonsteroidal anti-inflammatory drugs (NSAID), particularly acetylsalicylic acid (ASA), reduce the risk of colorectal neoplasia^9,10^ but with possible adverse events^11^. Indeed, a safe and effective CRC chemoprevention agent in average-risk population would help reducing the incidence of colorectal neoplasia.

Statins, inhibitors of 3-hydroxy-3-methyl-glutaryl-coenzyme A reductase, are a widely used and well-tolerated class of drugs for the treatment of hypercholesterolemia. Previous studies indicate their possible role in cancer chemoprevention^12,13^, with controversial results^14–16^. In addition to their main effect on cholesterol synthesis, statins may cause a number of other pleiotropic effects that may influence tumorigenesis, such as antioxidant activity, effects on cell adhesion, or angiogenesis^17^. *In vitro*, statins have shown anti-proliferative and pro-apoptotic effects on human CRC cell lines, and also in tumour xenograft models^18,19^. Studies analysing the effects of exposure to statins on the prevention or prognosis of colorectal neoplasia have shown controversial results, which have been proposed to be due to heterogeneity amongst drugs, or to effects restricted to some subgroup of patients^14–16,20^.

In this observational study we have analysed a population-based health records database aiming to examine the association between statins, their subtypes and pattern of use, and CRC risk.

## Methods

### Data source

Subjects were selected from the Information System for Development of Primary Care Research (SIDIAP) database (www.sidiap.org)^21^, which comprises clinical information routinely collected by primary care professionals of the Catalan Institute of Health. This database includes information from 5.8 million people in Catalonia (almost 80% of the population) that have ever contacted the public health system since 2005. The data retrieved included routine clinical data, such as diagnoses and health measurements, and was linked to information on dispensed prescriptions generated by pharmacies’ claims for reimbursement by the Catalan Health System. Drugs were coded according to the Anatomical Therapeutic Chemical (ATC) classification system^22^, and the date and quantity of the drug withdrawn from the pharmacy were recorded. Irreversible encoding of patient identifiers ensured anonymization of the information in the SIDIAP study database. The quality of SIDIAP data has been previously documented, and it has been used to study the epidemiology of health outcomes^23^.

All procedures performed in the study involving data from human participants were in accordance with the ethical standards of the institutional research committee, and with the 1964 Helsinki Declaration and its later amendments or comparable ethical standards. No informed consent was requested to the participating individuals, since this study was based on anonymized data routinely collected. No variables with potential to identify specific individuals were retrieved. The study protocol was approved by the Ethics Committee for Clinical Research of IDIAP Jordi Gol and all applicable regulatory requirements were fulfilled. The study was registered in ENCePP database with code EUPAS12697.

### Study design

**Figure 1:**
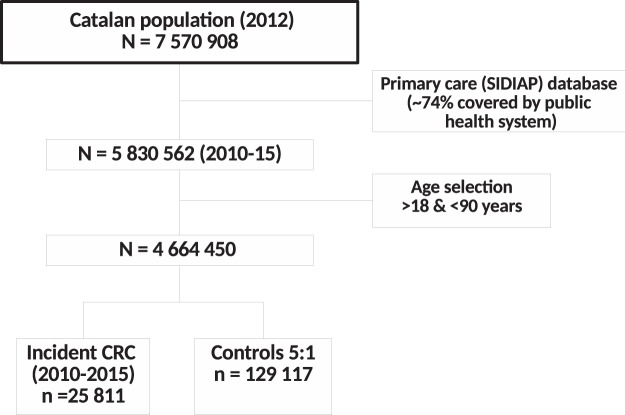
Population flowchart and study design. SIDIAP includes subjects that have interacted with the Catalan public health system (~74% of the total Catalan population). The study selected all CRC patients in the period 2010–2015 aged 18 to 90 years. Cases were incident diagnosis of colon or rectum (ICD-10 codes C18, C19, and C20). For each case, 5 matched controls of the same sex, age ± 5 years and health area were selected and assigned the case diagnosis date as index date for exposure assessment. Appendix cancer (C18.1) cases were excluded.

A population-based case-control study nested within the cohort of subjects receiving primary care from the Institut Català de la Salut was conducted. The flow chart of the study is described in Fig. 1. The cohort of subjects registered in SIDIAP with at least one healthcare interaction in last 3 years (n = 5 830 562) was limited to adult population, aged 18 to 90 years (n = 4 664 450). Cases identified with a recorded incident diagnosis of colon or rectum (codes C18, C19, and C20 of the International Classification of Diseases 10th Revision [ICD-10]) within the period January 1, 2010 to December 31, 2015 were identified. Those cases with a diagnosis of appendix cancer (C18.1) were excluded to avoid the inclusion of carcinoid tumours which are more frequent in that location.

A random stratified selection of controls was obtained using the same SIDIAP database. For each case, five controls were randomly selected from the set of all subjects in the database without prior CRC and alive at the time of diagnosis of the case, with the same age (±5 years) and sex and living in the same region defined by the primary healthcare centre catchment area. For cases, the disease onset date, defined as the earliest CRC diagnosis date registered, was set as the index date. For controls, the index date of their matched case was applied. Information regarding comorbidities and drug use was truncated to that recorded prior to the index date for cases and controls.

To assess if the codes used for case identification were reliable and exhaustive, and also to obtain an indirect measure of the external validity of our sample, we estimated the expected number of incident CRC cases in the population covered by the database according to cancer registries in Catalonia that contribute to the International Agency for Research on Cancer (IARC) publication Cancer Incidence in Five Continents (CI5) Volume XI^24^. The age-specific incidence rates of colon and rectum cancer estimated in the Tarragona and Girona cancer registries for 2012 were downloaded from https://ci5.iarc.fr. The age-specific rates were averaged over registries and summed for colon and rectum, then multiplied by the total Catalan population for the same age groups and period downloaded from https://www.idescat.cat. The population was corrected multiplying by 0.8 to account the average SIDIAP cumulative coverage on the studied period. Supplementary Fig. 1 shows that the number and age-sex distribution of the cases observed was similar to those expected.

### Exposure variables

Patients were classified as exposed to statins if they had retrieved at least one dispensation with an Anatomical Therapeutic Chemical code beginning with “C10AA”; otherwise, they were classified as unexposed. We also obtained exposure data for nonsteroidal anti-inflammatory drugs (NSAIDs) including aspirin (M01A, N02BA, N02BB, and B01AC06) as they potentially could confound the association between statin use and cancer risk, and other lipid lowering drugs (C10AB, C10AC, C10AD, C10AX). Daily defined doses (DDD) for each dispensed prescription were calculated by multiplying the container pills by dose (in mg) and dividing by the World Health Organization defined DDD (in mg) for each individual drug^22^. The average dose for each of these duration categories was established, dividing the sum of DDD by the interval length. Finally, to measure the effect of timing of exposure, we compared non-users to the subjects exposed exclusively 1 month to 5 years before the index date (short exposure) and the subjects exposed throughout the period of more than 5 years before the index date (long exposure). We chose ≥5 years as a cut-off point of long term, according to the mean of follow-up and statin use in randomized controlled trials.

In order to compare the results with previous studies, statins were classified as lipophilic (atorvastatin, fluvastatin, lovastatin, simvastatin) or hydrophilic (pravastatin and rosuvastatin) and, by effectiveness in lowering LDL cholesterol levels, as low-potency (fluvastatin, lovastatin, pravastatin, simvastatin) and high-potency (atorvastatin and rosuvastatin)^25^.

### Confounders

The potential confounders identified a priori for this analysis were age, sex, socioeconomic status, region, year, body mass index (BMI), tobacco, alcohol, comorbidity conditions and NSAID use^3,9,10^. Socioeconomic status was evaluated using the MEDEA socioeconomic deprivation score^26^, which was divided into quintiles for the analysis. Chronic comorbidity conditions considered for multivariable adjustment included those associated with CRC in the data: hypertension, hyperuricemia, diabetes, osteoarthritis and spondyloarthropathy, chronic lower respiratory diseases, extrapyramidal and movement disorders, episodic and paroxysmal disorders, mental and behavioural disorders, chronic kidney disease, heart failure, cerebrovascular disease, liver disease, insomnia, osteoporosis, peptic ulcer, inflammatory bowel disease. Conditions clearly defined as indications of statins (dyslipidaemia and cardiovascular disease) were not considered for adjustment, since they are in the explored causal path.

### Statistical analysis

Odds ratios (OR) and 95% confidence intervals (95% CI) were calculated from unconditional logistic regression models. We compared the effect of no use to any use of drug and assessed effects of dose (DDD) and duration of statin use. We also explored the effect of the type of the statin (potency and lipophilicity). Subgroup analyses were performed according to sex, age groups, NSAID use and cancer location (colon or rectum). Missing data for body mass index (BMI) was imputed using the prediction of a linear model according to age, sex and, outcome status (63% had complete data). To avoid models with many parameters, an adjustment score was built from the predictions of a logistic regression model for CRC that included all potential confounding covariates, without selection strategies. This adjustment score was efficient to render all potential confounders non-significantly associated to CRC. All logistic regression models included the adjustment score. P-values were derived from likelihood ratio tests. Statistical analysis was carried out using R statistical software (R Foundation for Statistical Computing, Vienna, Austria).

## Results

### Baseline characteristics and statin exposure

Characteristics of the study population according to statin use are presented in Table 1. During the study period there were 25 811 CRC cases which were matched by sex, age at time of index date (±5 years), and healthcare region to 129 117 controls. A total of 66 372 (42.8%) subjects were ever users of 3-Hydroxy-3-methylglutaryl-coenzyme A reductase inhibitors at the index date, 55 008 (42.6%) were controls and 11 364 (44.0%) CRC cases. Statin users were older with only 3.2% being less than age 55 years at entry (median age 74 years for users and 67 years for non-users). Statin consumption was associated with age, male sex, data of entry in the cohort, higher BMI, former smoking, severe alcohol consumption and, higher NSAID prescription (Table 1). Statin users were more likely to have comorbidities (see Supplementary Table 1).

**Table 1:** Characteristics of the study according to statin use (controls only, N = 129117).

Characteristic	Statin non-users		Statin users		OR^a^	95% CI	P-value (trend)
	n	%	n	%			
**Age**							
18–55 years	13076	17.6	1778	3.2	1		
55–65 years	17948	24.2	8793	16.0	3.59	3.40–3.80	
65–75 years	17945	24.2	17560	31.9	7.17	6.79–7.56	
75–85 years	18065	24.4	20912	38.0	8.50	8.06–8.97	
85–95 years	7075	9.5	5965	10.8	6.23	5.86–6.62	<0.0001
**Sex**							
Male	43557	58.8	33112	60.2	1		
Female	30552	41.2	21896	39.8	0.93	0.91–0.96	<0.0001
**Year of entry** (**years**)							
2010	12871	17.4	7743	14.1	1		
2011	12913	17.4	8426	15.3	1.11	1.07–1.16	
2012	12884	17.4	9582	17.4	1.24	1.19–1.29	
2013	12754	17.2	10297	18.7	1.33	1.28–1.38	
2014	12858	17.4	10500	19.1	1.38	1.33–1.44	
2015	9829	13.3	8460	15.4	1.43	1.37–1.49	<0.0001
**Body Mass Index**							
≤25 kg/m^2^	12217	16.5	8769	15.9	1		
25.1–30.0 kg/m^2^	47902	64.6	29691	54.0	0.91	0.88–0.94	
>30 kg/m^2^	13990	18.9	16548	30.1	1.67	1.61–1.73	<0.0001
**Tobacco** ^**b**^							
Non-smoker	35247	61.3	31761	61.0	1		
Current smoker	10875	18.9	6608	12.7	0.94	0.91–0.98	
Former smoker	11392	19.8	13701	26.3	1.42	1.38–1.47	
**Alcohol** ^**b**^							
None/mild	30591	63.1	30927	64.5	1		
Moderate	16524	34.1	16010	33.4	1.02	0.99–1.05	
Severe	1387	2.9	1045	2.2	0.89	0.81–0.96	0.004
**NSAIDs**							
Non-users	31529	42.5	8525	15.5	1		
Users	42580	57.5	46483	84.5	3.61	3.51–3.71	<0.0001

^a^Adjusted for age and sex.

^b^Variables with missing data.

NSAID: Nonsteroidal anti-inflammatory drugs

The most frequent statin used was simvastatin (n = 48 907, 31.6% of all subjects) followed by atorvastatin (n = 25 198, 16.3% of all subjects) (Table 2). Of the 66 372 subjects ever exposed to any statin, 32 559 (49.1.5%) were long term users of statins (≥5 years). There were 22 995 (34.7%) individuals that were ever exposed to more than one different statin. The most common multiple exposure was simvastatin and atorvastatin, followed by simvastatin and pravastatin (Table 2).

**Table 2:** Statin use and CRC risk.

		Controls		CRC cases		OR^a^	95% CI	P-value
		n	%	n	%			
Overall effect	Non-user	74109	57.4	14447	56.0	1		0.11
	Statin user	55008	42.6	11364	44.0	0.98	0.95–1.01	
Duration^b^	Non-user	74109	57.4	14447	56.0	1		0.0002
	<5 years	22515	17.4	4568	17.7	1.11	1.04–1.18	
	≥5 years	27002	20.9	5557	21.5	0.96	0.93–1.00	
Cumulative dose	Non-user	74109	57.4	14447	56.0	1		0.06
	<709 DDD	18344	14.2	3784	14.7	1.00	0.96–1.04	
	709–2370 DDD	18307	14.2	3817	14.8	0.98	0.94–1.02	
	>2370 DDD	18357	14.2	3763	14.6	0.95	0.91–0.99	
Lipophilicity	Non-user	74109	57.4	14447	56.0	1		0.38
	Lipophilic	45456	35.2	9418	36.5	0.98	0.95–1.01	
	Hydrophilic	2844	2.2	567	2.2	0.96	0.87–1.05	
	Both	6708	5.2	1379	5.3	0.96	0.91–1.03	
Statin potency	Non-user	74109	57.4	14447	56.0	1		0.13
	Low potency	33139	25.7	6821	26.4	0.98	0.94–1.01	
	High potency	7900	6.1	1678	6.5	1.01	0.96–1.07	
	Both	13969	10.8	2865	11.1	0.96	0.91–1.00	
Type of statin	Simvastatin	40584	31.4	8323	32.2	0.97	0.94–0.99	0.02
	Atorvastatin	20839	16.1	4359	16.9	0.99	0.96–1.03	0.75
	Pravastatin	7964	6.2	1635	6.3	0.98	0.93–1.04	0.48
	Fluvastatin	3447	2.7	698	2.7	0.98	0.90–1.06	0.59
	Lovastatin	3132	2.4	677	2.6	1.04	0.96–1.13	0.35
	Rosuvastatin	1915	1.5	372	1.4	0.92	0.82–1.03	0.16
	Pitavastatin	325	0.3	69	0.3	1.03	0.79–1.34	0.83
	Simvastatin + atorvastatin^c^	11353	8.8	2355	9.1	0.97	0.93–1.02	0.29
	Simvastatin + pravastatin^c^	3973	3.1	832	3.2	1.00	0.92–1.08	0.95

^a^Adjusted for age, sex, socioeconomic status, region, year, body mass index, smoking, alcohol, comorbidities and nonsteroidal anti-inflammatory drugs use.

^b^Variables with missing data.

^c^Non-users or users of only one of the two drugs as reference category.

A detailed table with the characteristics of the study population for cases of CRC and controls is presented in Supplementary Table 2. The environmental variables associated with CRC were lower BMI, former smoking and alcohol consumption. The median number of months on statin use was 58 months for both cases and controls.

### Statin use and colorectal cancer

There was no overall association of statin use with CRC risk (OR = 0.98; 95% CI: 0.95–1.01, P = 0.11) (Table 2). The analysis of duration of exposure showed a significant 11% increase of risk for exposures to statins shorter than 5 years, while the analysis of cumulated exposure as derived from sum of DDDs per subject was not significant. Moreover, the risk of CRC was similar in patients with current or former exposure, and also among those who stopped taking statins 6 months, 12 months and 36 months before the index date.

No differences were observed when statins were classified by their potency or lipophilicity. The analysis of specific statins did not show differential effects regarding CRC risk. Finally, as Supplementary Table 3 shows, there was no interaction according to age groups (P-value for interaction = 0.06) nor gender (P-value for interaction = 0.09). There was a significant interaction for NSAIDs exposure, so that exposure to was protective in NSAIDs users but increased risk in non-users (P-value for interaction <0.01).

### Statin use and rectal cancer

We performed a stratified analysis to determine whether CRC location influenced the effect of statins (Table 3). A statistically significant interaction of tumour location was observed (P < 0.001), with a significant reduction of 13% in the risk of rectal cancer (adjusted OR = 0.87; 95% CI: 0.81–0.92, P < 0.001), but not of colon cancer (adjusted OR 1.00; 95% CI: 0.97–1.03, P = 0.91). However, no consistent dose-effect was seen when analysing duration and dose. All types of statins showed similar significant associations for rectal cancer.

**Table 3:** Analyses of statins effect according to CRC location.

		Controls		Colon cases				P-value^b^	Rectal cases				P-value^b^	P-interaction^c^
		n	%	n	%	OR^a^	95% CI		n	%	OR^a^	95% CI		
Overall effect	Non-user	74109	57.4	11754	55.3	1		0.91	2693	58.9	1		<0.001	<0.001
	Statin user	55008	42.6	9488	44.7	1.00	0.97–1.03		1876	41.1	0.87	0.81–0.92		
Duration^d^	Non-user	74109	57.4	11754	55.3	1		0.001	2693	58.9	1			<0.001
	<5 years	22515	17.4	3813	18.0	0.99	0.95–1.03		755	16.5	0.96	0.82–1.11		
	≥5 years	27002	20.9	4636	21.8	0.98	0.95–1.02		921	20.2	0.85	0.79–0.93		
Cumulative dose	Non-user	74109	57.4	11754	55.3	1		0.10	2693	58.9	1		<0.001	
	<709 DDD	18344	14.2	3167	14.9	1.03	0.98–1.07		617	13.5	0.87	0.80–0.96		<0.001
	709–2370 DDD	18307	14.2	3202	15.1	1.01	0.97–1.06		615	13.5	0.85	0.78–0.93		
	>2370 DDD	18357	14.2	3119	14.7	0.96	0.92–1.01		644	14.1	0.87	0.80–0.96		
Lipophilicity	Non-user	74109	57.4	11754	55.3	1		0.94	2693	58.9	1		<0.001	<0.001
	Lipophilic	45456	35.2	7851	37.0	1.00	0.97–1.04		1567	34.3	0.88	0.82–0.94		
	Hydrophilic	2844	2.2	490	2.3	1.01	0.92–1.12		77	1.7	0.70	0.55–0.88		
	Both	6708	5.2	1147	5.4	0.99	0.92–1.05		232	5.1	0.87	0.76–1.00		
Statin potency	Non-user	74109	57.4	11754	55.3	1		0.28	2693	58.9	1		<0.001	<0.001
	Low potency	33139	25.7	5690	26.8	1.00	0.97–1.04		1131	24.8	0.87	0.81–0.94		
	High potency	7900	6.1	1414	6.7	1.05	0.99–1.12		264	5.8	0.86	0.75–0.98		
	Both	13969	10.8	2384	11.2	0.98	0.93–1.03		481	10.5	0.86	0.78–0.96		

^a^Adjusted for age, sex, socioeconomic status, region, year, body mass index, smoking, alcohol, comorbidities and NSAID use.

^b^P-value for trend.

^c^P-value for the interaction between colon and rectum.

^d^Variables with missing data.

### Analysis of other lipid lowering drugs

A significantly increased risk of CRC was observed for subjects exposed to bile acid sequestrants (OR 1.33; 95% CI: 1.12–1.57, p = 0.001), but no significant association with CRC was seen for exposures to neither fibrates nor nicotinic acid, independently on location, statin potency or dose (Supplementary Table 4).

## Discussion

In this study, based on the SIDIAP database, which is representative of the Catalan population, we found a high prevalence of exposure to statins, above 40% both in cases and controls. We observed that exposure to statins was not significantly associated with the overall risk of CRC, but might be associated to a modestly reduced rectal cancer risk. For colon cancer or the combination of colon and rectal cancer (colorectal), there was no decrease in risk associated with statin use. There were no consistent associations observed for duration and cumulated dose of statin exposure and rectal cancer, while the protective association was similar for diverse statin types. Besides, exposure to acid bile sequestrants showed an increase of risk of 33%.

Previous case-control and cohort studies have suggested that statins could play a role in cancer chemoprevention^12,13^. However, data from clinical trials have not confirmed the protective effect seen in observational studies^14^. Recently, two meta-analyses and one systematic review including 40, 42, and 59 individual studies, respectively^14,15,20^, reported a modest reduction in risk of CRC among statin users. In contrast, previous studies that linked pharmacy and cancer registry databases found no associations between statin use and CRC risk^27–34^. Our study adds further evidence to the lack of a relevant effect of statins on the risk of incident CRC, supporting that any effect, if present, is of marginal magnitude. Our observation of a significant 11% increase of risk related to statin exposures shorter than 5 years is isolated, not found in other analysis such as the one exploring tumour location, and thus is of uncertain value.

Liu *et al*.^15^ showed in a stratified analyses a significant decreased association of risk in rectal cancer and for lipophilic statins, but this was limited to observational studies, and not when data was obtained from clinical trials. Our study has also observed that statin use may be selectively associated with reduced risk of rectal cancer. The reasons for this disparity in site association are unclear. Though colon and rectum are very similar at the molecular level^35^, environmental factors^36,37^ such as tobacco and physical activity^38^ differ in their role in their carcinogenesis. Moreover, there are clear differences among these two cancer locations regarding anatomic, embryologic, and physiologic differences^39,40^.

The analysis of the other lipid-lowering agents was to rule out confusion by indication, and it detected that bile acid sequestrants increased the risk of CRC, but all other lipid-lowering agents were not associated with CRC, similarly to statins. This finding was unexpected; while bile acid sequestrants are mainly used for the treatment of dyslipidaemia as they reduce low-density lipoprotein cholesterol^41^, they are less used than statins due to their poor tolerability (only 0.7% of our population was exposed), so that generally they are only prescribed to patients intolerant to statins or with severe dyslipidaemia. The role of bile acids on colorectal carcinogenesis has been widely studied^42^, and one trial had already reported a potential increase of CRC for long term use of cholestyramine back in 1992^43^, but we have found no other references analysing the effect of these drugs on CRC, which may merit further research.

Potential limitations of this study, common to others using routinely collected data, include the lack of individual validation of exposure or cancer status. Nevertheless, SIDIAP has been widely used for other epidemiologic studies, and previous validation studies have shown that the collected information is reliable regarding disease coding^21,23,44^. Regarding cancer location, we found a high proportion (72%) of cases classified as “colon not specified” (C18.9), and a lower number of rectal cancers than expected (18% observed vs 48% expected^45^). However, the total number of cases combining colon and rectum was consistent with those expected based on the incidence data published by the Catalan cancer registries (Supplementary Fig. 1)^45^. While it is plausible that some rectal cancers might be coded as “colon cancer not specified”, we can assume that the specificity of the rectal cancer location should be high. Misclassification was probably independent of statin use, and although might reduce statistical power for the analysis of rectal cancer, any bias, if existent, should be towards the null hypothesis. Regarding exposure, we used actually dispensed prescriptions at pharmacies. Though there is no way to prove that dispensed prescriptions were actually consumed, data on dispensed drugs is more reliable than electronic prescriptions, which may overestimate exposure at the expense of prescriptions that are never dispensed at pharmacies. Another limitation was that we could not adjust for some risk factors for CRC like physical activity, diet or family history of CRC, because these data were not available or did not reach usable quality. Finally, we have observed in our population that a lower BMI was associated with CRC. This is a typical finding in case-control studies, because the BMI data is registered close to the cancer diagnosis, in order to objectivize weight loss caused by the CRC as a part of the diagnostic procedures, while controls may have BMI recorded more often when obesity is requiring a clinical intervention by primary care physician.

The strengths of this study include the large sample size, and the high representativeness of the population, since SIDIAP includes data on roughly 80%% of the Catalan population. Because SIDIAP contains data collected in routine practice conditions, the likelihood of observer bias is minimized. The use of electronic medical records and invoicing databases allowed us to overcome memory bias. Despite the study period was limited to 2010–2015, individual medication data was available from 2005 onwards, which allowed us to study a long period of exposure, ensuring a minimum of 5 years before the CRC diagnosis. This is of paramount importance when studying diseases with long latency such as cancer, and, in fact short exposure time is a major criticism to statin randomized trials.

In conclusion, this study adds further evidence about the lack of a relevant association between statin utilization and risk of incident CRC. While we found no association between the use of statins and overall colorectal cancer risk, the suggestive evidence of a decrease in risk for rectal cancer requires further research.

## Supplementary information

Supplementary Figure and Tables

## Data Availability

The datasets generated and analysed during the current study are not publicly available due to restrictions imposed by the data provider (the Information System for Development of Primary Care Research, SIDIAP) but researchers interested can contact SIDIAP (www.sidiap.org) to propose a research project based on their database.
