# Supplementary material for: Statin use and the risk of colorectal cancer in a population-based electronic health records study
Source: Sci Rep. 2019 Sep 19;9:13560. doi: 10.1038/s41598-019-49877-5 (PMC6753123; doi:10.1038/s41598-019-49877-5)
Supplement: Supplementary file 1 — Supplementary Figure and Tables [file 41598_2019_49877_MOESM1_ESM.docx]

**Statin use and the risk of colorectal cancer in a population-based electronic health records study**

**Authors**

Gemma Ibáñez-Sanz^1,2,3,4^; Elisabet Guinó^1,3,4^; Caridad Pontes^5,6^; Mª Ángeles Quijada-Manuitt^7^; Luisa C de la Peña-Negro^1,2,3,8^; María Aragón^9^; Marga Domínguez^1^; Lorena Rodríguez-Alonso^2,3^; Alex Blasco^10^; Ana García-Rodríguez^8^; Rosa Morros^5,9,11*^; Victor Moreno^1,3,4,12^*

**Affiliations**

^1^Programa d’Analítica de Dades en Oncologia, Institut Català d’Oncologia, L’Hospitalet de Llobregat, Spain.

^2^Servei d’Aparell Digestiu, Hospital Universitari de Bellvitge, L’Hospitalet de Llobregat, Spain.

^3^Grup de Càncer Colorectal, Programa ONCOBELL, Institut d'Investigació Biomèdica de Bellvitge (IDIBELL), Hospitalet de Llobregat, Spain

^4^CIBER Epidemiologia i Salud Pública (CIBERESP), Madrid, Spain.

^5^Departament de Farmacologia, de Terapèutica i de Toxicologia, Universitat Autònoma de Barcelona, Barcelona, Spain

^6^Hospital de Sabadell, Institut Universitari Parc Taulí. Sabadell, Spain;

^7^Departament de Patologia i Terapèutica Experimental. Unitat Docent Campus de Bellvitge, Universitat de Barcelona, L’Hospitalet de Llobregat, Spain

^8^Servei d’Aparell Digestiu, Hospital de Viladecans, Viladecans, Spain.

^9^Institut d’Investigació en Atenció Primària Jordi Gol, Universitat Autònoma de Barcelona, Barcelona, Spain.

^10^Servei d’Aparell Digestiu, Hospital de Moisés Broggi, Sant Joan Despí, Spain.

^11^Institut Català de la Salut (ICS), Spain

^12^Departament de Ciències Clíniques, Facultat de Medicina i Ciències de la Salut, Universitat de Barcelona, Barcelona, Spain.

***Correspondence to:**

Rosa Morros

IDIAP Jordi Gol

Gran Via de les Corts Catalanes 587

08007 Barcelona, Spain.

E-mail: rmorros@idiapjgol.org

Victor Moreno

Cancer Prevention and Control Unit, Catalan Institute of Oncology

Av. Gran Via, 199-203

08908 L’Hospitalet de Llobregat, Barcelona, SPAIN

Email: v.moreno@iconcologia.net

Telephone: +932607186

Fax: +932607956

**
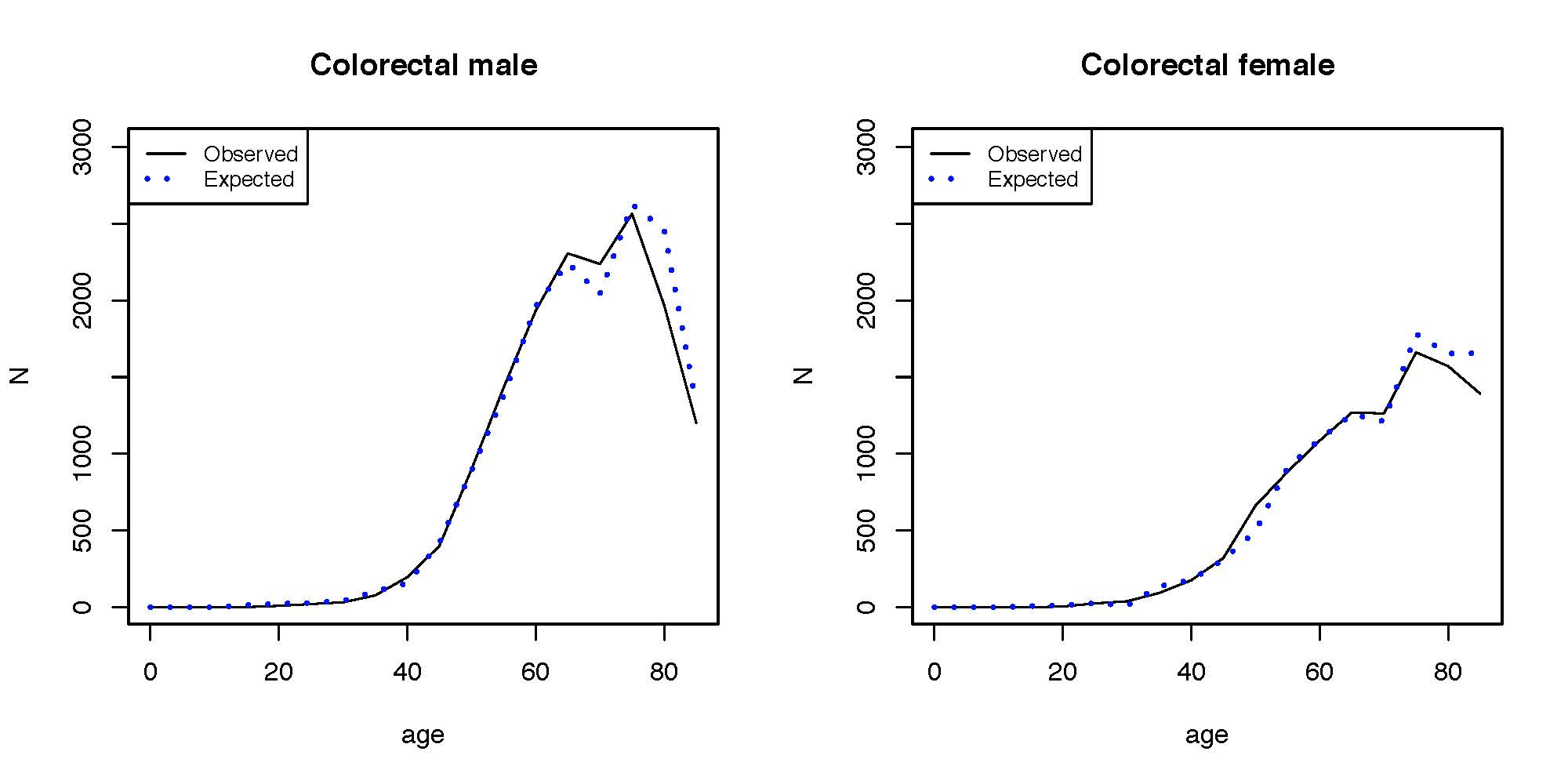
**

## Supplementary Figure 1.

## Incidence of CRC in Tarragona and Girona cancer registries compared to incidence registered in SIDIAP

Observed CRC cases (continuous line) in the SIDIAP database, compared to the expected number according to the average incidence in the Tarragona and Girona cancer registries. Expected numbers (dotted line) have been corrected to show that SIDIAP covers 74% of the Catalan population.

## Supplementary Table 1.

## Comorbidities of the study participants according to statin use (controls only, n= 129117)

| **Characteristic** | **Statin non-users** | | **Statin users** | | **OR^1^** | **95% CI** | **P-value** | **OR^2^** | **95% CI** | **P-value** |
| --- | --- | --- | --- | --- | --- | --- | --- | --- | --- | --- |
|  | **n** | **%** | **n** | **%** |  |  |  |  |  |  |
| Liver disease | 240 | 0.3 | 115 | 0.2 | 0.58 | 0.47 - 0.73 | 0.002 | **0.59** | **0.45 - 0.79** | **<0.001** |
| Dementia | 2481 | 3.3 | 2616 | 4.8 | 0.86 | 0.81 - 0.91 | <0.001 | **0.79** | **0.73 - 0.85** | **<0.001** |
| Extrapyramidal and movement disorders | 875 | 1.2 | 908 | 1.7 | 0.96 | 0.87 - 1.05 | 0.37 | 1.02 | 0.91 - 1.15 | 0.76 |
| Cancer non-CRC | 3388 | 4.6 | 2972 | 5.4 | 1.05 | 1.00 - 1.11 | 0.05 | 0.97 | 0.91 - 1.04 | 0.43 |
| Osteoporosis | 1426 | 1.9 | 1499 | 2.7 | 1.07 | 1.00 - 1.16 | 0.06 | 1.03 | 0.94 - 1.13 | 0.55 |
| Inflammatory bowel disease | 378 | 0.5 | 319 | 0.6 | 1.13 | 0.97 - 1.32 | 0.12 | 1.07 | 0.89 - 1.30 | 0.47 |
| Peptic ulcer | 2686 | 3.6 | 2728 | 5.0 | 1.21 | 1.14 - 1.28 | <0.001 | 0.99 | 0.93 - 1.07 | 0.86 |
| Insomnia | 3775 | 5.1 | 4114 | 7.5 | 1.28 | 1.22 - 1.34 | <0.001 | 1.02 | 0.96 - 1.08 | 0.57 |
| Chronic respiratory diseases | 9661 | 13.0 | 10384 | 18.9 | 1.29 | 1.25 - 1.33 | <0.001 | **1.06** | **1.02 - 1.11** | **<0.001** |
| Menopause^3^ | 2114 | 2.9 | 1565 | 2.8 | 1.31 | 1.22 - 1.41 | <0.001 | 1.08 | 0.99 - 1.17 | 0.09 |
| Episodic and paroxysmal disorders (Epilepsy) | 574 | 0.8 | 602 | 1.1 | 1.34 | 1.19 - 1.51 | 0.001 | 1.04 | 0.90 - 1.21 | 0.59 |
| Mental and behavioural disorders | 19528 | 26.4 | 17505 | 31.8 | 1.39 | 1.35 - 1.42 | <0.001 | **1.09** | **1.06 - 1.13** | **<0.001** |
| Osteoarthritis and spondyloarthropathy | 19730 | 26.6 | 21685 | 39.4 | 1.44 | 1.41 - 1.48 | <0.001 | **1.07** | **1.03 - 1.10** | **<0.001** |
| Colorectal polyps | 1295 | 1.7 | 1629 | 3.0 | 1.61 | 1.49 - 1.73 | <0.001 | **1.20** | **1.10 - 1.32** | **<0.001** |
| Hyperuricemia | 3103 | 4.2 | 4508 | 8.2 | 1.77 | 1.68 - 1.86 | <0.001 | 0.99 | 0.93 - 1.05 | 0.76 |
| Heart failure | 2111 | 2.8 | 3929 | 7.1 | 1.80 | 1.70 - 1.90 | <0.001 | 1.04 | 0.96 - 1.11 | 0.33 |
| Chronic kidney disease | 3198 | 4.3 | 6147 | 11.2 | 1.95 | 1.87 - 2.04 | <0.001 | **1.23** | **1.16 - 1.30** | **<0.001** |
| Hypertensive diseases | 28976 | 39.1 | 36715 | 66.7 | 2.47 | 2.41 - 2.54 | <0.001 | **1.57** | **1.53 - 1.62** | **<0.001** |
| Cerebrovascular disease | 3064 | 4.1 | 7037 | 12.8 | 2.55 | 2.44 - 2.67 | <0.001 | **2.40** | **2.27 - 2.53** | **<0.001** |
| Diseases of arteries, arterioles and capillaries | 1557 | 2.1 | 4240 | 7.7 | 3.08 | 2.90 - 3.28 | <0.001 | 1.04 | 0.96 - 1.11 | 0.33 |
| Diabetes mellitus | 8579 | 11.6 | 17611 | 32.0 | 3.11 | 3.02 - 3.20 | <0.001 | **2.40** | **2.32 - 2.49** | **<0.001** |
| Ischemic heart diseases | 1612 | 2.2 | 9545 | 17.4 | 7.91 | 7.48 - 8.35 | <0.001 | **7.43** | **6.98 - 7.91** | **<0.001** |

A patient with two or comorbidities would be counted in multiple rows.

^1^ Adjusted for age and sex.

^2^ Adjusted for age, sex, and all the other comorbidities in order to explore the net association for each comorbidity.

^3^ Only women.

**Supplementary Table 2.**

**Characteristics of the study participants and association of every risk factor with CRC**

| **Characteristic** | | **Controls** | | **CRC Cases** | | **OR^3^** | **95% CI** | **OR^4^** | **95% CI** | **P-trend** |
| --- | --- | --- | --- | --- | --- | --- | --- | --- | --- | --- |
|  |  | **n** | **%** | **n** | **%** |  |  |  |  |  |
| Age^1^ | |  |  |  |  |  |  |  |  |  |
|  | 18-55 years | 14854 | 11.5 | 2966 | 11.5 | 1 |  | 1 |  | <0.001 |
|  | 55-65 years | 26741 | 20.7 | 5344 | 20.7 | 1 | 0.95 - 1.05 | 0.95 | 0.90 - 1.00 |  |
|  | 65-75 years | 35505 | 27.5 | 7096 | 27.5 | 1 | 0.95 - 1.05 | 0.90 | 0.86 - 0.95 |  |
|  | 75-85 years | 38977 | 30.2 | 7796 | 30.2 | 1 | 0.96 - 1.05 | 0.89 | 0.85 - 0.93 |  |
|  | 85-95 years | 13040 | 10.1 | 2609 | 10.1 | 1 | 0.95 - 1.06 | 0.89 | 0.84 - 0.94 |  |
| Sex^1^ | |  |  |  |  |  |  |  |  |  |
|  | Male | 76669 | 59.4 | 15331 | 59.4 | 1 |  | 1 |  | <0.001 |
|  | Female | 52448 | 40.6 | 10480 | 40.6 | 1 | 0.97 - 1.03 | 1.07 | 1.04 - 1.10 |  |
| Deprivation Index Score MEDEA^1^ | |  |  |  |  |  |  |  |  |  |
|  | Urban 1st quintile -least deprived | 21335 | 17.8 | 4143 | 17.8 | 1 |  | 1 |  | 0.24 |
|  | Urban 2nd quintile | 19684 | 16.4 | 3805 | 16.3 | 1 | 0.95 - 1.04 | 0.97 | 0.93 - 1.02 |  |
|  | Urban 3rd quintile | 19250 | 16.0 | 3682 | 15.8 | 0.98 | 0.94 - 1.03 | 0.96 | 0.91 - 1.00 |  |
|  | Urban 4th quintile | 18681 | 15.6 | 3599 | 15.4 | 0.99 | 0.94 - 1.04 | 0.96 | 0.91 - 1.01 |  |
|  | Urban 5th quintile -most deprived | 16007 | 13.3 | 3075 | 13.2 | 0.99 | 0.94 - 1.04 | 0.95 | 0.90 - 1.00 |  |
|  | Rural | 25147 | 20.9 | 5000 | 21.5 | 1.02 | 0.98 - 1.07 | 0.98 | 0.93 - 1.03 |  |
| Body Mass Index | |  |  |  |  |  |  |  |  |  |
|  | ≤25 kg/m^2^ | 20986 | 16.3 | 4610 | 17.9 | 1 |  | 1 |  | <0.001 |
|  | 25.1-30.0 kg/m^2^ | 77593 | 60.1 | 14914 | 57.8 | 0.87 | 0.84 - 0.91 | 0.93 | 0.90 - 0.97 |  |
|  | >30 kg/m^2^ | 30538 | 23.7 | 6287 | 24.4 | 0.94 | 0.90 - 0.98 | 0.91 | 0.87 - 0.95 |  |
| Tobacco^2^ | |  |  |  |  |  |  |  |  |  |
|  | Non-smoker | 67008 | 61.1 | 13515 | 59.2 | 1 |  | 1 |  | <0.001 |
|  | Current smoker | 17483 | 16.0 | 3646 | 16.0 | 1.03 | 0.99 - 1.08 | 0.95 | 0.91 - 1.00 |  |
|  | Former smoker | 25093 | 22.9 | 5676 | 24.9 | 1.13 | 1.09 - 1.18 | 1.07 | 1.03 - 1.12 |  |

**Supplementary Table 2 (cont.)**

| **Characteristic** | | **Controls** | | **CRC Cases** | | **OR^3^** | **95% CI** | **OR^4^** | **95% CI** | **P-trend** |
| --- | --- | --- | --- | --- | --- | --- | --- | --- | --- | --- |
|  |  | **n** | **%** | **n** | **%** |  |  |  |  |  |
| Alcohol^2^ | |  |  |  |  |  |  |  |  |  |
|  | None/mild | 61518 | 63.8 | 12590 | 61.9 | 1 |  | 1 |  |  |
|  | Moderate | 32534 | 33.7 | 7077 | 34.8 | 1.07 | 1.03 - 1.11 | 1.08 | 1.04 - 1.12 |  |
|  | Severe | 2432 | 2.5 | 680 | 3.3 | 1.37 | 1.25 - 1.50 | 1.35 | 1.23 - 1.48 |  |
| Comorbidity index | |  |  |  |  |  |  |  |  |  |
|  | Low | 70789 | 54.8 | 12771 | 49.5 | 1 |  | 1 |  | <0.001 |
|  | Medium | 17720 | 13.7 | 3531 | 13.7 | 1.10 | 1.05 - 1.14 | 1.14 | 1.10 - 1.18 |  |
|  | High | 40608 | 31.5 | 9509 | 36.8 | 1.31 | 1.27 - 1.35 | 1.26 | 1.21 - 1.30 |  |
| NSAIDs | |  |  |  |  |  |  |  |  |  |
|  | Non-users | 40054 | 31.0 | 7643 | 29.6 | 1 |  | 1 |  | 0.53 |
|  | Users | 89063 | 69.0 | 18168 | 70.4 | 1.07 | 1.04 - 1.10 | 0.99 | 0.96 - 1.02 |  |
| Statins | |  |  |  |  |  |  |  |  |  |
|  | Non-users | 74109 | 57.4 | 14447 | 56.0 | 1 |  | 1 |  | 0.23 |
|  | Users | 55008 | 42.6 | 11364 | 44.0 | 1.06 | 1.03 - 1.09 | 0.98 | 0.95 - 1.01 |  |

^1^Variable used for matching.

^2^Variable with missings.

^3^Adjusted for age and sex

^4^Adjusted by all variables shown in the table.

## Supplementary Table 3.

## Analysis of statins effect on CRC stratified according to age, sex, and NSAID use

|  | | **Controls** | | **Cases** | | **OR^1^** | **95% CI** | **P-value** | **P-interaction** |
| --- | --- | --- | --- | --- | --- | --- | --- | --- | --- |
|  | | **n** | **%** | **n** | **%** |  |  |  |  |
| **Age** | |  |  |  |  |  |  |  |  |
|  | 18-55 years | 1778 | 12 | 416 | 14 | 1.07 | 0.95 - 1.2 | 0.28 | 0.06 |
|  | 55-65 years | 8793 | 32.9 | 1866 | 34.9 | 0.98 | 0.91 - 1.04 | 0.46 |  |
|  | 65-75 years | 17560 | 49.5 | 3661 | 51.6 | 1.00 | 0.95 - 1.06 | 0.86 |  |
|  | 75-85 years | 20912 | 53.7 | 4219 | 54.1 | 0.96 | 0.92-1.01 | 0.14 |  |
|  | 85-95 years | 5965 | 45.7 | 1202 | 46.1 | 0.96 | 0.88 - 1.05 | 0.34 |  |
|  |  |  |  |  |  |  |  |  |  |
| **Sex** | |  |  |  |  |  |  |  |  |
|  | Male | 33112 | 43.2 | 6809 | 44.4 | 0.95 | 0.91 - 0.98 | 0.004 | 0.09 |
|  | Female | 21896 | 41.7 | 4555 | 43.5 | 1.01 | 0.97 - 1.05 | 0.69 |  |
| **NSAID non-user** | |  |  |  |  |  |  |  | <0.001 |
|  | Statin non-user | 31529 | 78.7 | 5744 | 75.2 | 1 |  | 0.05 |  |
|  | Statin user | 8525 | 21.3 | 1899 | 24.8 | 1.06 | 1.00 - 1.13 |  |  |
| **NSAID user** | |  |  |  |  |  |  |  |  |
|  | Statin non-user | 42580 | 47.8 | 8703 | 47.9 | 1 |  | 0.003 |  |
|  | Statin user | 46483 | 52.2 | 9465 | 52.1 | 0.95 | 0.92 - 0.98 |  |  |

^1^Adjusted for age, sex, socioeconomic status, region, year, body mass index, smoking, alcohol and comorbidities.

NSAID: Nonsteroidal anti-inflammatory drugs

## Supplementary Table 4.

## Lipid modifying agents and CRC risk

|  | | **Controls** | | **Cases** | | **OR^1^** | **95% CI** | **P-value** |
| --- | --- | --- | --- | --- | --- | --- | --- | --- |
|  | | **n** | **%** | **n** | **%** |  |  |  |
| **Fibrates** | |  |  |  |  |  |  |  |
|  | Non-user | 121926 | 94.4 | 24362 | 94.4 | 1 |  | 0.06 |
|  | User | 7191 | 5.6 | 1449 | 5.6 | 0.94 | 0.89 - 1.00 |  |
| **Bile acid sequestrants** | |  |  |  |  |  |  |  |
|  | Non-user | 128455 | 99.5 | 25632 | 99.3 | 1 |  | 0.001 |
|  | User | 662 | 0.5 | 179 | 0.7 | 1.33 | 1.12 - 1.57 |  |
| **Nicotinic acid and derivatives** | |  |  |  |  |  |  |  |
|  | Non-user | 129054 | 100 | 25800 | 100 | 1 |  | 0.50 |
|  | User | 63 | 0 | 11 | 0 | 0.81 | 0.43 - 1.54 |  |
| **Any lipid modifying agent** | |  |  |  |  |  |  |  |
|  | Non-user | 121339 | 94 | 24205 | 93.8 | 1 |  | 0.34 |
|  | User | 7778 | 6.0 | 1606 | 6.2 | 0.97 | 0.92 - 1.03 |  |

^1^Adjusted for age, sex, socioeconomic status, region, year, body mass index, smoking, alcohol, comorbidities and nonsteroidal anti-inflammatory drugs use.
